# Influence of Surface Pits on Roughness in Single-Point Diamond Turning of Particle-Reinforced Polymer Composites: A Theoretical and Experimental Study

**DOI:** 10.3390/mi17080915

**Published:** 2026-07-29

**Authors:** Zhihao Zhang, Yazhou Zhang, Chunlei He

**Affiliations:** 1 Tianjin Key Laboratory of Equipment Design and Manufacturing Technology, Tianjin University, Tianjin 300354, China; zhzhang_jx@tju.edu.cn; 2 School of Computer Science and Technology, Tianjin University, Tianjin 300354, China; yzhou_zhang@tju.edu.cn

**Keywords:** ultra-precision cutting, single-point diamond turning, surface roughness prediction, pit formation mechanism, particle-reinforced polymer matrix composite

## Abstract

In this study, the surface pit formation mechanism during single-point diamond face turning of a particle-reinforced polymer matrix composite (PRPMC) was investigated using finite element simulations. Based on the identified pit formation mechanisms, a surface roughness prediction model was developed and validated through face turning experiments. The results show that particle fracture and interfacial debonding are the primary origins of surface pits. Increasing cutting depth promotes the transition from particle fracture to interfacial debonding, leading to larger pits and higher surface roughness. A rake angle of −15° and a larger tool nose radius improve surface quality by suppressing pit formation and plastic side flow. The proposed model accurately predicts the effects of cutting depth, rake angle, and tool nose radius on surface roughness, with a maximum prediction error of 9.20% and an average error of 4.66%. This study provides a theoretical basis for surface roughness prediction and process parameter optimization in single-point diamond face turning of PRPMCs.

## 1. Introduction

Particle-reinforced polymer matrix composites (PRPMCs) are an important class of engineering materials consisting of hard reinforcement particles embedded in a polymer matrix [1,2,3,4]. Owing to their high specific stiffness, excellent dimensional stability and tailorable multifunctional properties, PRPMCs have been widely used in aerospace, optics, precision manufacturing, electronic packaging and other engineering applications [5,6,7,8]. However, the pronounced mismatch in the mechanical properties of the reinforcement particles and the polymer matrix results in highly heterogeneous deformation and complex material removal behavior during cutting, posing significant challenges to achieving high-quality machined surfaces [9,10,11]. Since surface roughness is a key indicator of machining quality, understanding its formation mechanism is essential for improving the ultra-precision machining performance of PRPMCs.

Previous studies on PRPMCs have primarily focused on their mechanical behavior and damage mechanisms under various loading conditions [12,13,14,15]. These investigations demonstrated that cutting parameters, tool geometry and particle characteristics strongly influence machining behavior and surface integrity. Finite element (FE) simulations have also been employed to investigate deformation and damage mechanisms during particle-reinforced composite machining [16,17,18,19]. Existing studies have shown that heterogeneous deformation of the matrix and reinforcement particles may induce local damage and surface defects during machining. However, the mechanisms governing surface pit formation and their influence on surface roughness remain poorly understood.

Ultra-precision cutting, particularly single-point diamond turning (SPDT), has been widely used for manufacturing high-quality surfaces on advanced materials owing to its excellent dimensional accuracy and repeatability [20,21,22,23,24,25]. Numerous surface roughness prediction models have been developed for metallic materials by considering factors such as tool geometry, elastic recovery and plastic side flow [26,27,28,29,30,31,32]. However, these models do not account for the surface pits generated by crystal fracture and particle debonding in PRPMCs. Consequently, their applicability to PRPMC machining remains limited.

In this study, a barium sulfate PRPMC is selected as the representative material. FE simulations are employed to investigate the mechanisms of surface pit formation during ultra-precision cutting. The effects of machining parameters on surface pit formation and surface roughness are analyzed, and a theoretical model for predicting surface roughness is established. Furthermore, ultra-precision cutting experiments using single-crystal diamond tools with different geometries are conducted to verify the FE simulation results and validate the proposed theoretical model.

## 2. Models and Experiments

### 2.1. FE Model

**Figure 1:**
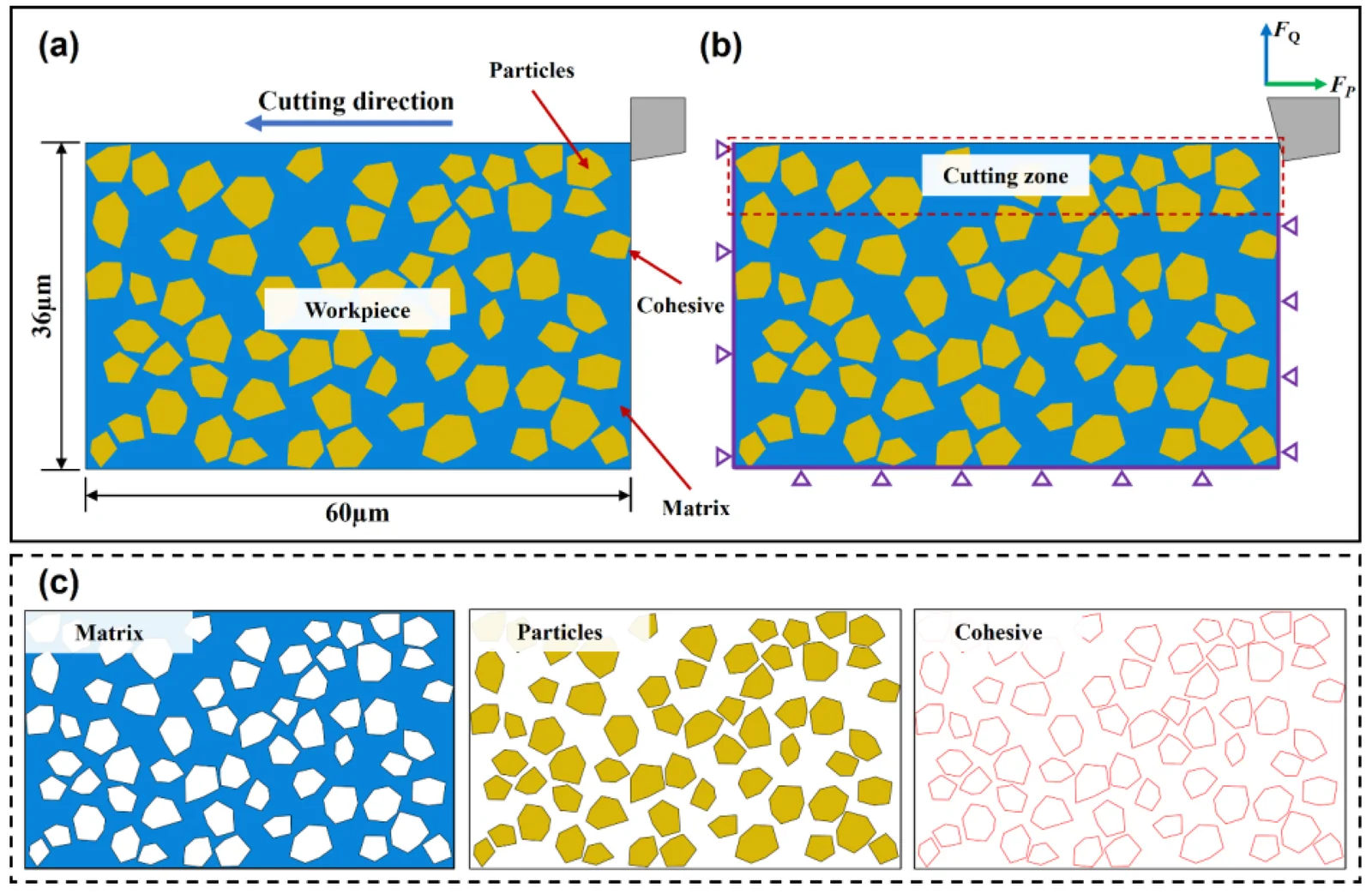
FE model of PRPMC cutting: (**a**) Cutting with a 0° rake angle tool. (**b**) Cutting with a −15° rake angle tool. (**c**) Microstructural configuration of the PRPMC workpiece.

Figure 1 illustrates the FE model established in ABAQUS 2022 to simulate the ultra-precision cutting process of the PRPMC. The model consists of a PRPMC workpiece and a diamond cutting tool. The workpiece comprises a 60 μm × 36 μm polymer matrix containing polygonal particles with an average size of 5 μm. The particle volume fraction was set to 60%, consistent with that of the experimental specimens. The diamond tools are modeled as rigid bodies with rake angles of 0° and −15°, a flank angle of 9°, and a cutting edge radius of 30 nm. The FE model is established to represent the local material removal behavior during single-point diamond face turning. Accordingly, the simulated cutting configuration is consistent with the experimental face turning process and focuses on the material removal mechanisms in the vicinity of the cutting edge. The barium sulfate crystal particles and polymer matrix were discretized using four-node plane strain reduced-integration elements CPE4R, while cohesive elements were employed to model the particle–matrix interfaces. A global mesh size of 200 nm was adopted throughout the model. Mesh sensitivity analysis confirmed that the selected mesh provided sufficient numerical accuracy for the present simulations. Displacement constraints were applied to the lateral and bottom boundaries to simulate workpiece clamping. The model length was 60 μm, whereas the cutting simulation was terminated at a cutting distance of 28 μm. Consequently, a sufficiently large material domain remained around the cutting zone throughout the simulation, ensuring that the influence of boundary constraints on the local stress field was negligible. The cutting depth (*ap*) is set to 2 μm, with the diamond tool cutting at a speed of 400 mm/s. Although the cutting speed varies with the cutting radius during face turning, previous studies have demonstrated that variations in cutting speed within the range used in single-point diamond turning have a negligible effect on the resulting surface roughness [33]. Therefore, a representative constant cutting speed of 400 mm/s was adopted in the present FE simulations. The coefficient of friction between the tool and the sample is taken as a constant of 0.3 [34], corresponding to a friction angle of 16.7°. Table 1 provides the detailed simulation parameters.

Different constitutive models are utilized for the matrix, particles, and the interface between the matrix and particles. The Johnson–Cook (J-C) constitutive model coupled with the J-C damage description characterizes the plastic deformation behavior of the polymer matrix under significant strains. Table 2 provides the corresponding J-C model parameters, where *A*, *B*, *C*, *m*, and *n* represent the initial yield strength, strain hardening modulus, strain rate sensitivity coefficient, thermal softening index, and strain hardening coefficient, respectively. The failure parameters *d*_1_–*d*_5_ represent the failure coefficients of the matrix and are used to predict the ductile damage of the matrix. Table 3 lists the physical parameters of the matrix, including density, elastic modulus, and Poisson’s ratio. Table 4 presents the parameters of the brittle fracture model for crystal particles. The matrix–particle interface is characterized using a cohesive element coupled with a secondary nominal stress damage criterion (Quads Damage) to effectively capture crystal particle debonding and interfacial failure. Table 5 provides detailed cohesive element parameters, where *k_n_* and *k_t_* denote the normal and tangential stiffness of the interface, respectively, while *t_n_* and ${\bar{t}}_{t}$ represent the normal and tangential nominal stresses.

The FE model employs polygonal crystal particles and assumes homogeneous material properties. Previous studies have demonstrated that such simplified microstructural representations can effectively capture the dominant mechanisms of matrix deformation, particle fracture, and debonding in heterogeneous particulate composites [42,43]. Therefore, these simplifications are not expected to affect the fundamental mechanisms governing surface pit formation identified in this study. It should be noted, however, that the present FE model is primarily intended to investigate the fundamental mechanisms of particle removal and the relative effects of cutting parameters, rather than to quantitatively reproduce the exact dimensions and morphology of individual surface pits. In actual PRPMCs, variations in particle morphology and particle size distribution may influence local stress concentrations, leading to differences in pit dimensions and local damage evolution.

### 2.2. Theoretical Model

**Figure 2:**
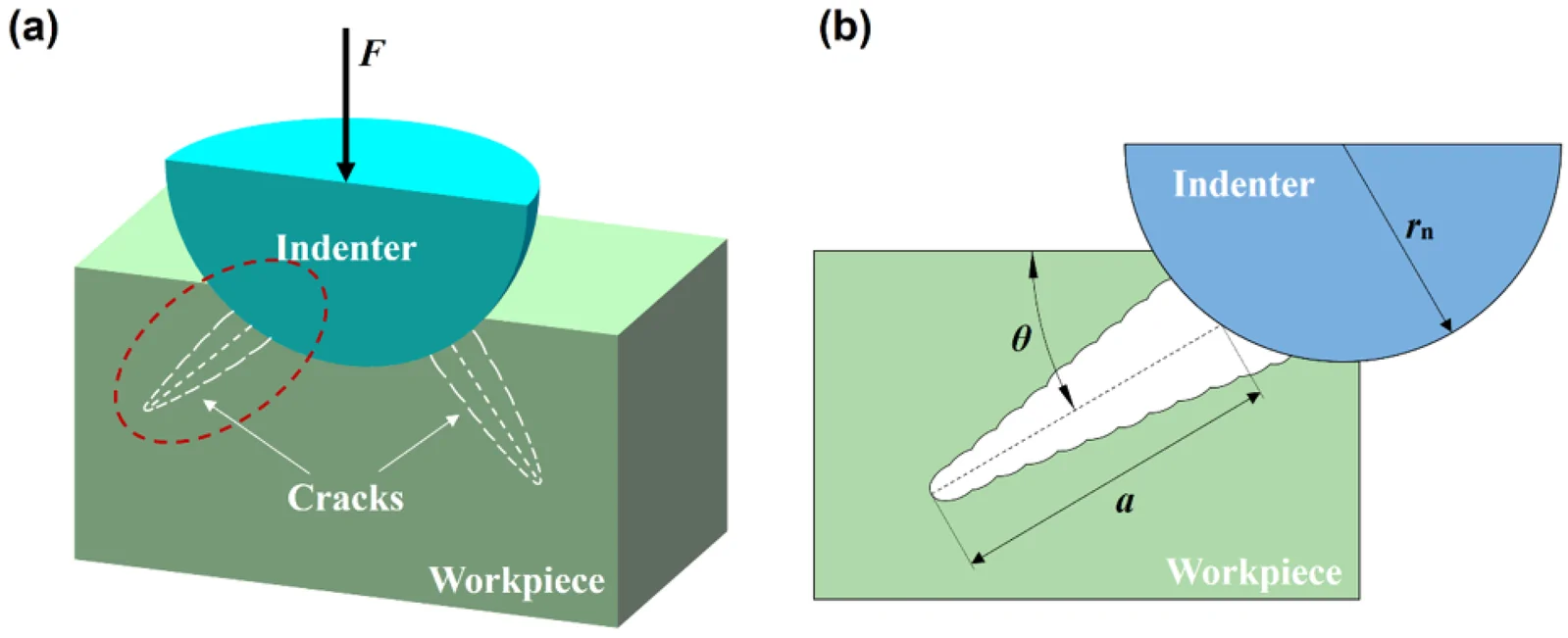
Schematic diagram of indentation fracture: (**a**) Distribution of cracks. (**b**) Length and orientation of a single crack.

The cutting edge of the diamond cutting tool is not perfectly sharp; it possesses a finite edge radius, denoted as *r*_n_. Consequently, the rounded cutting edge is represented as a spherical indenter, and the indentation fracture approach is employed to simulate the compressive interaction between the tool and crystal particles during the cutting process. Figure 2 illustrates the schematic of the indentation fracture model. As shown in Figure 2a, a spherical indenter with an applied load *F* induces crack formation in the workpiece. One of the cracks highlighted by the red circle is magnified in Figure 2b, where the primary crack exhibits a length *a* and forms an angle *θ* with the free surface.

**Figure 3:**
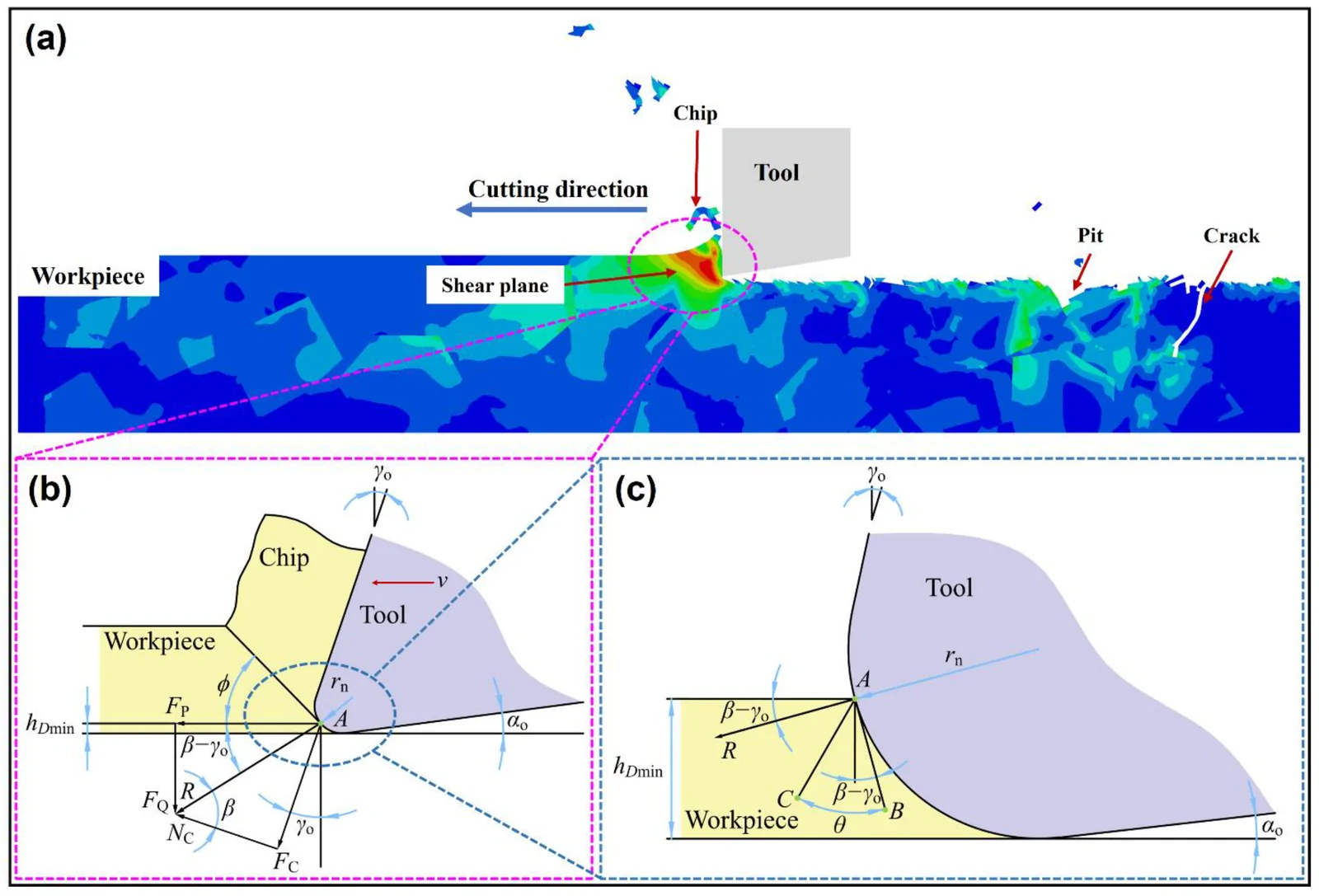
Force analysis of the cutting process and analysis of the primary crack angle: (**a**) FE simulation of cutting with a tool featuring a 0° rake angle. (**b**) Force analysis of the cutting process based on Merchant’s cutting theory. (**c**) Analysis of primary crack angles in the cutting process.

Unlike simple indentation fracture models, the cutting process involves more complex forces. Applying Merchant’s cutting theory [42] and the minimum undeformed chip thickness theory [28] to analyze the forces during cutting, Figure 3 illustrates the force conditions experienced by the tool during the cutting process. Figure 3a shows the FE simulation of cutting with a tool featuring a 0° rake angle. The shear plane formed during the cutting process can be observed, along with the occurrence of transgranular fracture and interfacial debonding in the crystal particles. In Figure 3b, material processed above the corresponding critical point *A* accumulates to form the chip, whereas below point *A*, the processed material undergoes elastic–plastic deformation to form the machined surface.

Analyzing the force conditions at critical point *A*, the resultant cutting force *R* acting on the workpiece is decomposed into the horizontal force *F*_P_ and the normal force *F*_Q_. The normal force *N*_C_ exerted by the rake face on the workpiece and the friction force *F*_C_ are translated to critical point *A*. Since the cutting edge radius *r*_n_ is extremely small, the moment generated by this translation is neglected. Neglecting the friction between the flank face and the machined surface, the resultant cutting force acting on the workpiece is considered to be equal in magnitude and opposite in direction to the resultant of the compressive and frictional forces exerted by the chip on the rake face. In Figure 3b, *F*_C_ and *N*_C_ denote the friction force and compressive force exerted by the chip on the rake face, respectively. The resultant of *F*_C_ and *N*_C_ is equal and in the same direction as the resultant cutting force *R*:

(1) $$R=\sqrt{{F_{P}}^{2}+{F_{Q}}^{2}}=\sqrt{{F_{C}}^{2}+{N_{C}}^{2}},$$

where the friction force *F*_C_ and the compressive force *N*_C_ satisfy the relationship

(2) $$\tan\beta =\mu =\frac{F_{C}}{N_{C}},$$

where *β* is the friction angle; *μ* is the coefficient of friction between the tool and the chip.

During the cutting process, crystal particles are subjected to both compressive and shear stresses, resulting in a composite fracture mode of type I–II for which the equivalent stress intensity factor criterion is employed [43,44]:

(3) $$\frac{{K_{I}}^{2}+{K_{II}}^{2}}{{K_{\mathrm{IC}}}^{2}+{K_{IIC}}^{2}}=1,$$

where *K*_I_ and *K*_II_ denote the Mode I and Mode II stress intensity factors, respectively; *K*_IC_ and *K*_IIC_ represent the Mode I and Mode II fracture toughness, respectively. Assuming that the unmachined material extends to infinity, the Mode I and Mode II stress intensity factors at the tip of a semi-infinite crack are given by [45]:

(4) $$K_{I}=1.1215\sigma \sqrt{\pi a}\sin^{2}\theta ,$$

(5) $$K_{II}=1.1215\sigma \sqrt{\pi a}\sin\theta \cos\theta ,$$

where *σ* denotes the normal stress. According to the maximum circumferential stress criterion [46], the relationship between Mode II fracture toughness and Mode I fracture toughness is as follows:

(6) $$K_{IIC}=0.866K_{IC}$$

Substituting Equations (4)–(6) into Equation (3) yields the simplified criterion:

(7) $${K_{I}}^{2}+{K_{II}}^{2}=1.75{K_{IC}}^{2}$$

The angle *θ* between the primary crack and the free surface is calculated as

(8) $$\sin^{2}\theta =\frac{1.39{K_{\mathrm{IC}}}^{2}}{\sigma^{2}\pi a},$$

where the primary crack length a is calculated as

(9) $$a={\left(\frac{\chi \sqrt{{F_{P}}^{2}+{F_{Q}}^{2}}}{K_{\mathrm{IC}}}\right)}^{\frac{2}{3}},$$

where *χ* is a material property related to the Poisson’s ratio ν:

(10) $$\chi \left(\nu \right)={\left\{\frac{3}{4}{\left[3{\left(1-2\nu \right)}^{2}\left(1-\nu^{2}\right)/32\pi \right]}^{3}\right\}}^{\frac{1}{2}}$$

Substituting Equation (9) into Equation (8), the angle *θ* between the primary crack and the free surface is calculated as:

(11) $$\sin^{2}\theta =\frac{1.39{K_{\mathrm{IC}}}^{\frac{8}{3}}}{\sigma^{2}\pi \chi^{\frac{2}{3}}R^{\frac{2}{3}}}$$

Enlarging the image within the blue circle in Figure 3b yields Figure 3c. Figure 3c illustrates the angle between the primary crack and the free surface during the cutting process, along with the crack length. The surface passing through critical point *A* and perpendicular to the resultant cutting force is defined as the free surface, denoted as *AB* in Figure 3c. From geometric relationships, the angle between the free surface *AB* and the normal force *F*_Q_ is given by *β − γ*_o_. The angle between the free surface and the primary crack is *θ*, while the angle between the primary crack and the normal direction is *θ* − (*β* − *γ*_o_).

**Figure 4:**
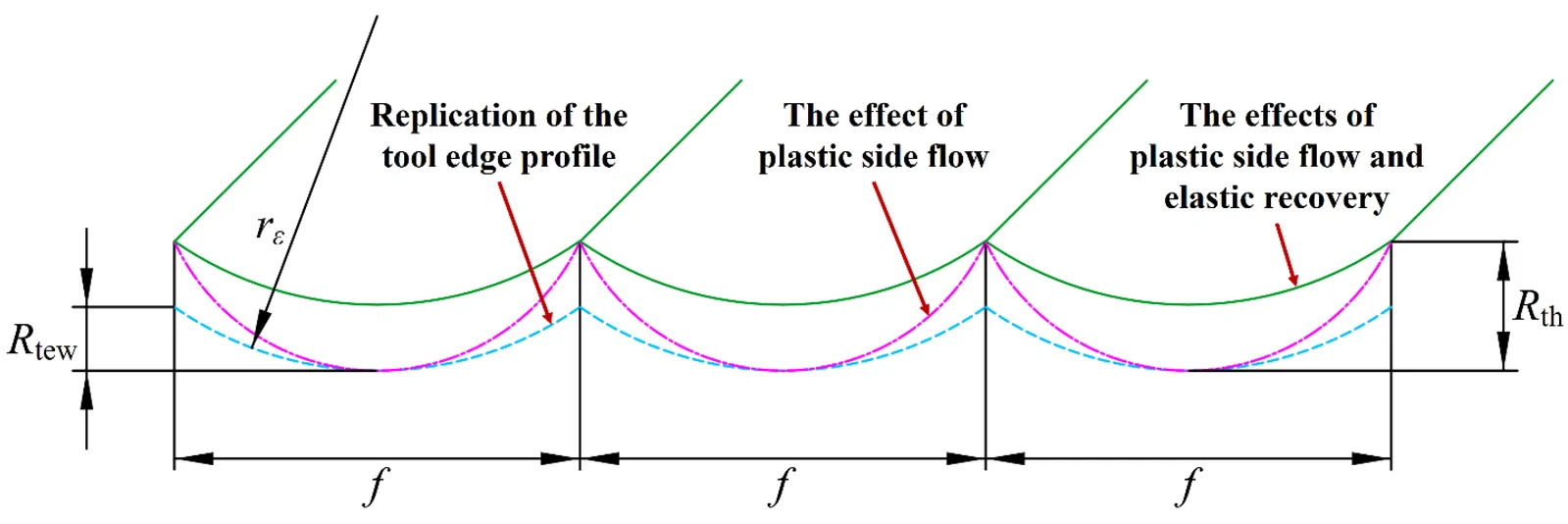
Residual profile in feed direction.

Figure 4 illustrates the basic single-point cutting model, where *f* denotes the feed rate and *r_ε_* represents the tool nose radius. Grzesik [27] considered the cutting edge replication effect and proposed that the height difference between the peak and valley of the residual profile along the feed direction corresponds to the theoretical residual height *R_tew_*, which can be calculated as follows:

(12) $$R_{tew}=\frac{f^{2}}{8r_{\varepsilon }}$$

On this basis, by considering the elastic recovery and plastic side flow components associated with the minimum undeformed chip thickness, the classical formula for surface roughness in ultra-precision cutting, known as the Spanzipfel equation, can be derived as follows:

(13) $$R_{th}=\frac{f^{2}}{8r_{\varepsilon }}+\frac{h_{D\min}}{2}\left(1+\frac{r_{\varepsilon }h_{D\min}}{f^{2}}\right),$$

where *h_D_*_min_ represents the minimum undeformed chip thickness. Liu et al. [28] derived the calculation formula for *h_D_*_min_ by analyzing the force conditions at the critical point *A*, as follows:

(14) $$h_{D\min}=\left[1-\frac{F_{Q}+\mu F_{P}}{\sqrt{\left({F_{P}}^{2}+{F_{Q}}^{2}\right)\left(1+\mu^{2}\right)}}\right]r_{n}$$

From Figure 3b, the relationship between *F*_Q_ and *F*_P_ is given by:

(15) $$\frac{F_{Q}}{F_{P}}=\tan\left(\beta -\gamma_{o}\right)$$

Substituting Equations (2) and (15) into Equation (14) gives the minimum undeformed chip thickness in terms of the tool rake angle:

(16) $$h_{D\min}=\left[1-\frac{\tan\left(\beta -\gamma_{o}\right)+\tan\beta }{\sqrt{\left(1+\tan^{2}(\beta -\gamma_{o})\right)\left(1+\tan^{2}\beta \right)}}\right]r_{n}$$

However, the Spanzipfel formula presented in Equation (13) does not account for the influence of the cutting depth on the plastic side flow component. In addition, according to the studies of Liu et al. [28] and He et al. [24] on the mechanism of plastic side flow, a smaller tool nose radius intensifies stress concentration during cutting, which in turn enhances the plastic side flow component. Therefore, based on Hertzian contact theory, the effect of the cutting depth on the plastic side flow component is incorporated, and a correction factor associated with the tool nose radius is introduced. The modified Spanzipfel formula is thus expressed as:

(17) $$R_{th\_rev}=\frac{f^{2}}{8r_{\varepsilon }}+\frac{h_{D\min}}{2}\left(1+k_{D}k_{r}\frac{r_{\varepsilon }h_{D\min}}{f^{2}}\right),$$

where *k*_D_ denotes the correction factor accounting for the effect of cutting depth, which is dimensionless, and *k*_r_ represents the correction factor associated with the tool nose radius, which is also dimensionless.

In Hertzian contact theory, the stress *p* is related to the indentation depth *d* of a rigid indenter as follows:

(18) $$p\propto {\left(c_{1}d-c_{2}\right)}^{0.5},$$

where *c*_1_ and *c*_2_ are constants related to the material properties. The maximum stress *p*_0_ is related to the curvature radius *r* of the rigid indenter as follows:

(19) $$p_{0}\propto r^{-0.5}$$

Therefore, in conjunction with Hertzian contact theory, the cutting depth correction factor *k*_D_ and the tool nose radius correction factor *k*_r_ are given by:

(20) $$k_{D}={\left(k_{1}ap+k_{2}\right)}^{0.5},$$

(21) $$k_{r}=k_{3}{r_{\varepsilon }}^{-1.5}+k_{4}{r_{\varepsilon }}^{-1},$$

where *k*_1_, *k*_2_, *k*_3_, and *k*_4_ are correction coefficients in relation to the workpiece material, which are configured as *k*_1_ = 5.01 μm^−1^, *k*_2_ = −4.10, *k*_3_ = 47,782.53 μm^1.5^, and *k*_4_ = −372.26 μm [24].

**Figure 5:**
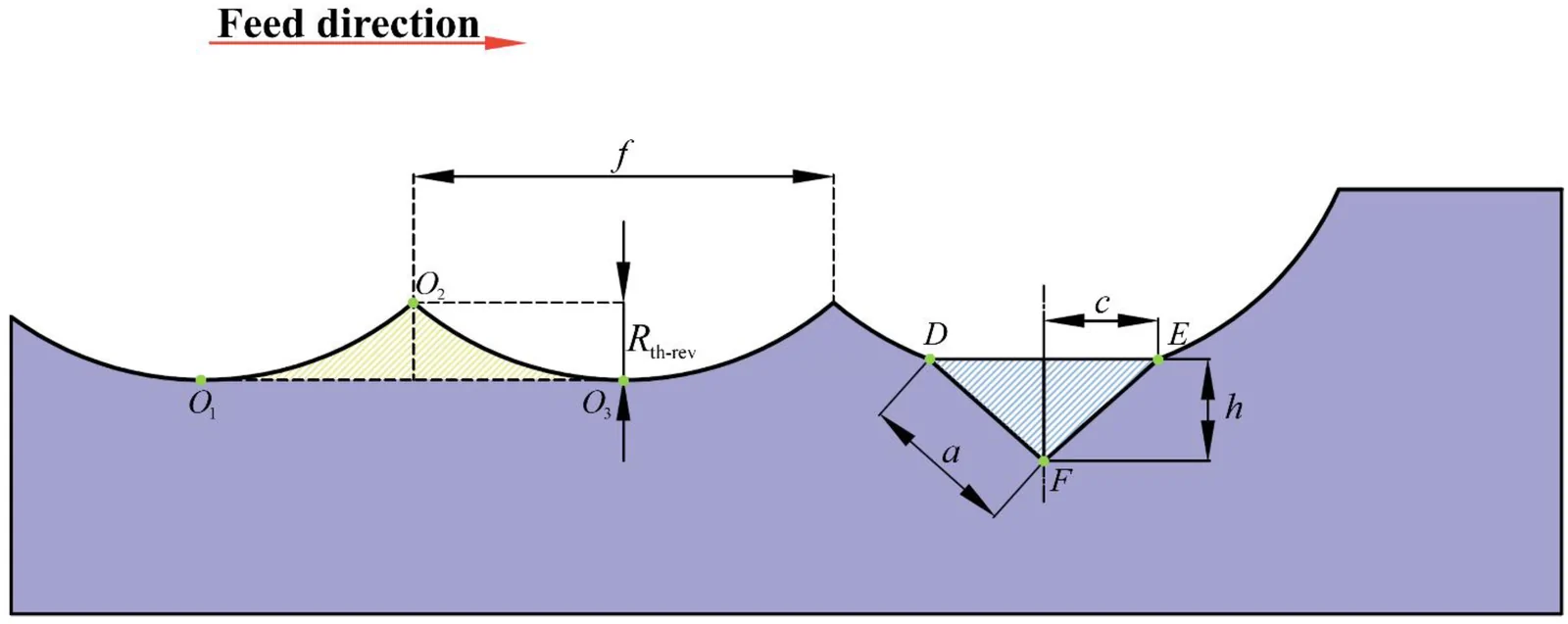
Residual profile in the feed direction with consideration of pit formation.

Figure 5 illustrates a surface roughness model accounting for pit effects. Based on the WLI observations (Figure 7) and the FE simulation results (Figure 9), the pits generated by transgranular fracture or interfacial debonding generally exhibit an approximately axisymmetric morphology, characterized by a deeper center and gradually shallower edges. To facilitate analytical derivation of pit volume and its contribution to surface roughness, each pit is therefore represented by an equivalent conical geometry, as shown by the blue shaded region *DEF* in Figure 5. It should be noted that this idealization is intended to capture the dominant volumetric contribution of pits to the arithmetic mean height *S_a_* rather than to reproduce the detailed irregular morphology of every individual pit. We denote the pit half-length as *c*, the pit depth as *h*, and the pit volume as *V*_1_. The pit half-length *c* and pit depth *h* are, respectively, represented by the crack length *a*:

(22) $$c=a\sin\left[\theta -\left(\beta -\gamma_{o}\right)\right]$$

(23) $$h=a\cos\left[\theta -\left(\beta -\gamma_{o}\right)\right]$$

The volume of the pit is expressed as:

(24) $$V_{1}=\frac{\pi }{3}c^{2}h=\frac{\pi }{6}a^{3}\sin\left\{2\left[\theta -\left(\beta -\gamma_{o}\right)\right]\right\}\sin\left[\theta -\left(\beta -\gamma_{o}\right)\right]$$

Let *l*_1_ be the sampling length in the feed direction and *l*_2_ be the sampling length in the cutting speed direction. The sampling area *S* is expressed as:

(25) $$S=l_{1}l_{2}$$

Assuming that the tool feeds *j* times within the sampling length *l*_1_ in the feed direction, forming *i* pits over the sampling area, *l*_1_ can be expressed in terms of *j* and *f* as:

(26) $$l_{1}=jf$$

The cutting edge profile is replicated onto the machined surface, and the unremoved material forms a feed direction residual profile with a volume denoted as *V*_2_. The cross-sectional area corresponding to this residual profile is represented by the yellow shaded region *O*_1_*O*_2_*O*_3_ in Figure 5. Since the feed per revolution is much smaller than the tool nose radius, the cross-section *O*_1_*O*_2_*O*_3_ can be approximated as a triangle, and the residual profile volume can thus be expressed as:

(27) $$V_{2}=\frac{f}{2}R_{th\_rew}l_{2}$$

The arithmetic mean height *S_a_* is expressed using the pit volume *V*_1_ and the residual volume *V*_2_ as follows:

(28) $$S_{a}=\frac{iV_{1}+\left(j-1\right)V_{2}}{S}$$

Substituting Equations (24)–(27) into Equation (28), the arithmetic mean height *S_a_* is expressed as:

(29) $$S_{a}=\frac{\pi ia^{3}\sin\left\{2\left[\theta -\left(\beta -\gamma_{o}\right)\right]\right\}\sin\left[\theta -\left(\beta -\gamma_{o}\right)\right]}{6jfl_{2}}+\frac{R_{th\_rev}}{2}$$

Substituting Equation (17) into Equation (29) yields the expressions for the residual height component *S_ath_* and the plastic side flow component *w*, respectively:

(30) $${S_{a}}_{th}=\frac{f^{2}}{16r_{\varepsilon }}$$

(31) $$w=\frac{h_{D\min}}{4}\left(1+k_{D}k_{r}\frac{r_{\varepsilon }h_{D\min}}{f^{2}}\right)$$

### 2.3. Experimental Setup

**Figure 6:**
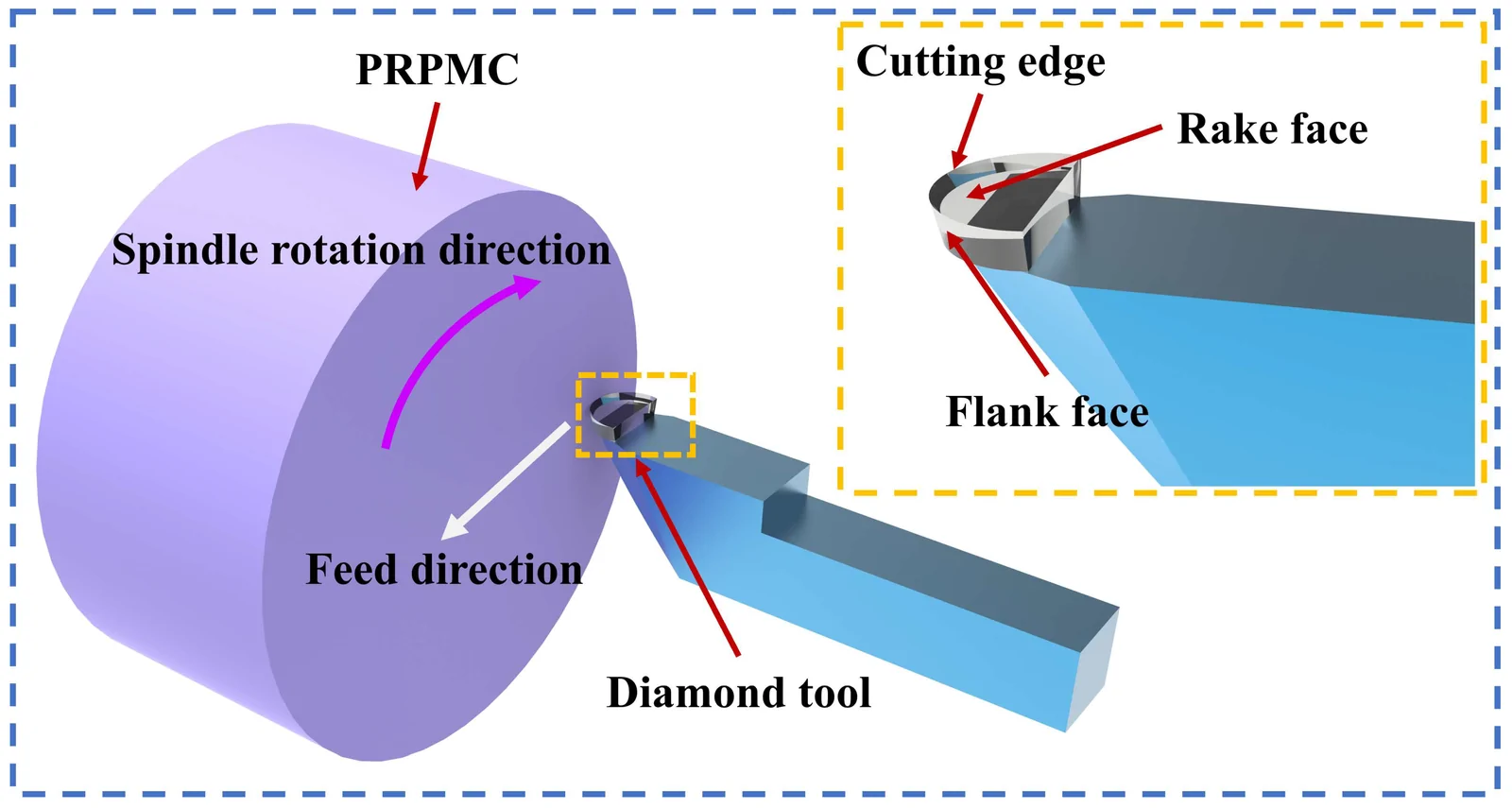
Experimental setup for face cutting of the PRPMC.

A series of face cutting experiments were conducted on an ultra-precision machine tool (Precitech Nanoform X). As illustrated in Figure 6, the workpiece was securely mounted on the rotating spindle using a precision vacuum chuck to ensure stable positioning during machining and rotated clockwise at 1000 rpm, while the tool was positioned on a linear axis to provide the feed. Dry cutting was employed throughout the experiments, and compressed air at a pressure of 0.3 MPa was continuously supplied to the cutting zone for chip removal and cooling. The workpiece material is a PRPMC with a diameter of 20 mm. The face cutting experiments were performed in two steps. In the first step, a polycrystalline diamond tool with a rake angle of 0°, a flank angle of 9°, and a tool nose radius of 10 mm was used for rough cutting to obtain a preliminary surface. In the second step, three natural single-crystal diamond tools were employed, with rake angles of 0° and −15°, a flank angle of 9°, tool nose radii of 1000 μm and 3000 μm, and a constant cutting edge radius of 30 nm. Table 6 lists the detailed parameters of the natural single-crystal diamond tools, and Table 7 presents the experimental conditions for the natural single-crystal diamond tool cutting tests. The experiments were designed to investigate the effects of cutting depth and tool geometry on the machined surface quality. The spindle speed and feed rate were kept constant throughout all experiments, while the cutting depth and tool geometry were varied according to the experimental conditions listed in Table 7.

The white light interferometer (WLI) provides a large field of view, enabling effective characterization of the overall surface topography. After the cutting experiments, the machined surfaces were immediately characterized using a CHOTEST SuperView W1 WLI (CHOTEST, Shenzhen, China). The measurement field of view was 800 μm × 800 μm, with a vertical scanning range of ±25 μm. During data processing, a Gaussian filter with a cutoff wavelength of 80 μm was applied to separate the roughness from waviness, followed by a 5 × 5 pixel Gaussian smoothing filter to suppress high-frequency measurement noise while preserving the intrinsic surface features. For each cutting condition, surface topography was measured at three different locations on the machined surface, and the *S_a_* value was obtained by averaging the three measurements. This multi-point measurement strategy improves the reliability of the surface roughness characterization.

## 3. Results and Discussion

### 3.1. Formation Mechanism of Surface Pit

**Figure 7:**
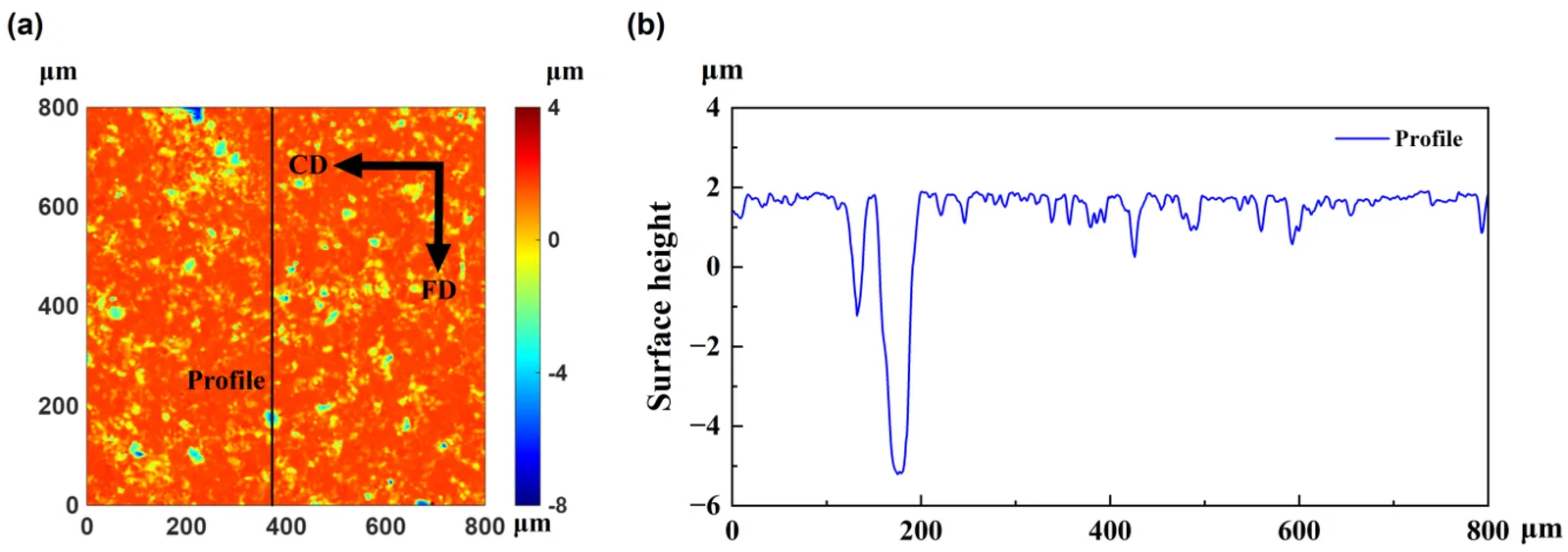
Surface topography of PRPMC after ultra-precision cutting (feed/cutting direction: FD/CD, cutting depth: 2 μm, tool rake angle: 0°, tool nose radius: 1000 μm): (**a**) Surface roughness measurement result via WLI (Sa 439.27 nm). (**b**) Extracted two-dimensional profile at x = 370 μm.

Figure 7 presents the surface topography of the PRPMC after ultra-precision cutting using a tool with a rake angle of 0° and a cutting depth of 2 μm. In Figure 7a, the feed direction is denoted as FD and the cutting direction as CD, with FD being perpendicular to CD. As illustrated in Figure 7a, numerous pits are observed on the machined surface of the PRPMC after ultra-precision cutting. The plastic side flow of the matrix has covered most of the tool marks on the machined surface, and the arithmetic mean height *S_a_* is 439.27 nm. As shown in Figure 7b, the two-dimensional profile extracted at *x* = 370 μm exhibits only slight fluctuations over the matrix regions, whereas pronounced undulations are observed at the particle locations. The pronounced undulations are attributed to particle debonding, whereas the minor undulations result from transgranular fracture of the particles.

**Figure 8:**
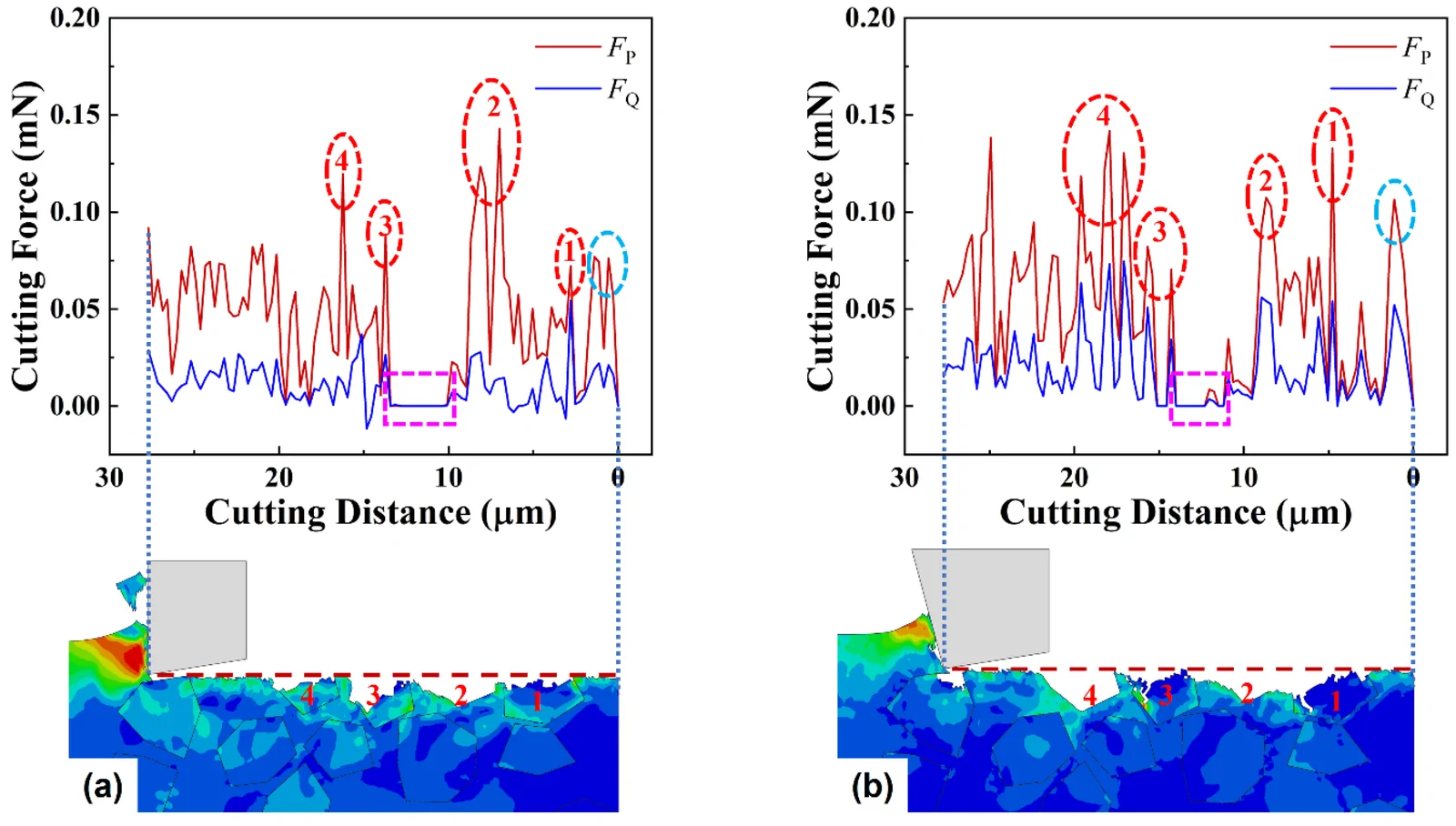
FE simulations of the cutting forces versus cutting distance at a cutting depth of 2 μm under different rake angles: (**a**) Rake angle: 0°. (**b**) Rake angle: −15°.

To elucidate the formation mechanism of surface pits, FE simulations were conducted. Figure 8 presents the variation in cutting forces during the cutting process for tools with two different rake angles at a cutting depth of 2 μm. At the cutting distance of 0 μm in Figure 8a,b, the tool engagement with the sample produces a small peak (indicated by the blue circles) in cutting force. Subsequently, larger cutting force peaks (highlighted by the red circles) are generated when cutting crystal particles, which are highlighted with red indices in the figure, whereas cutting the matrix results in smaller fluctuations in the cutting force. In addition, within the cutting distance range of 10–13 μm in Figure 8a,b, the cutting force is approximately zero (marked by the magenta rectangles), which is consistent with the phenomenon observed by Huang et al. [47]. This is attributed to debonding of crystal particle No. 2 during the cutting process, leading to an idle cutting condition where the cutting force drops to zero.

**Figure 9:**
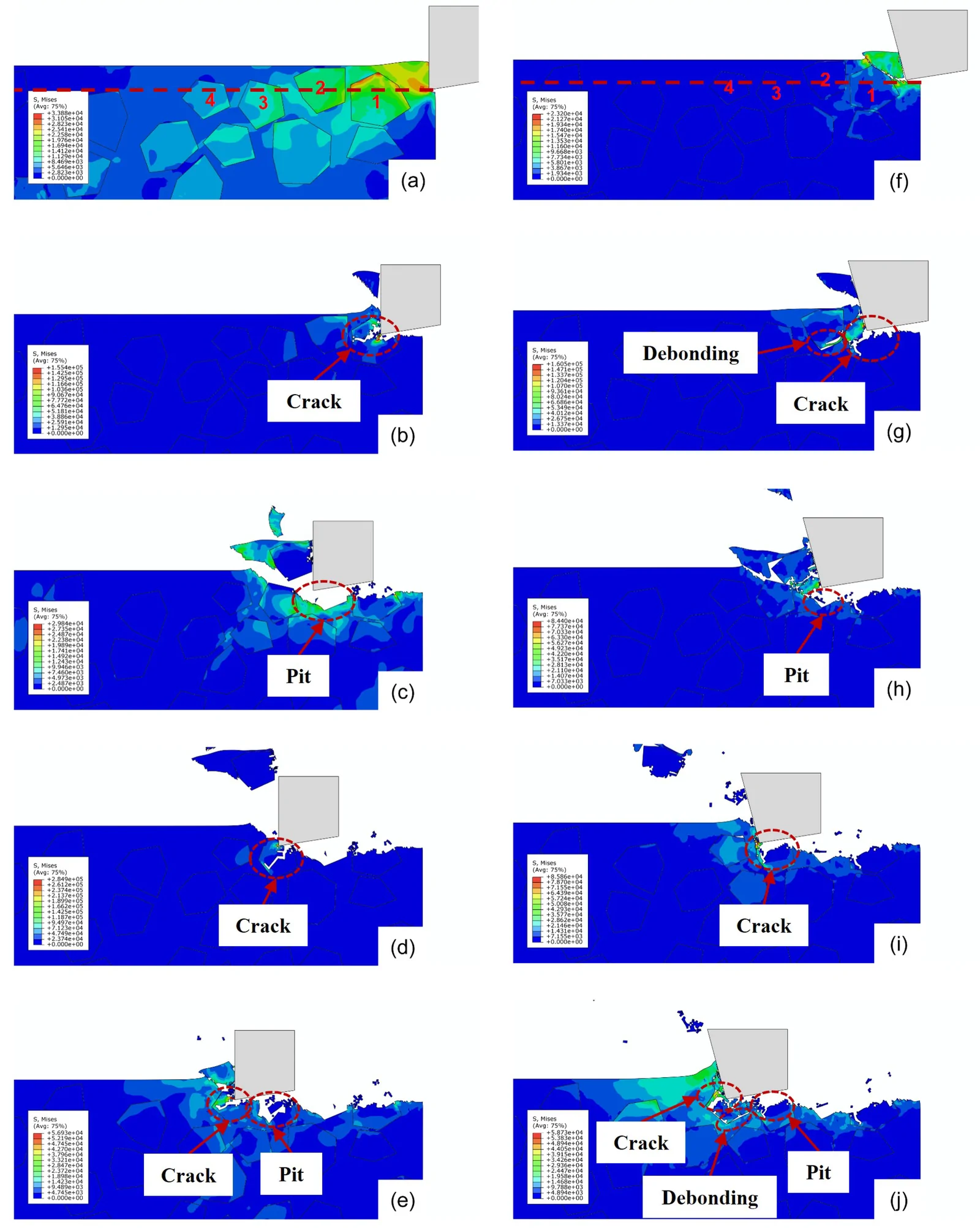
FE simulations of the cutting process at a cutting depth of 2 μm under different rake angles: (**a**–**e**) Rake angle: 0°. (**f**–**j**) Rake angle: −15°.

Figure 9a and Figure 9f show simulation at the commencement of cutting with a cutting depth of 2 μm, using tools with rake angles of 0° and −15°, respectively, where crystal particles numbered 1, 2, 3, and 4 are located along the cutting path. In Figure 9b,g, during the cutting of crystal particle No. 1, the cutting path passes near the maximum diameter of the crystal particle. Under the direct compression of the rake face, stress concentration occurs and leads to fracture. A portion of the fractured particles is crushed and expelled as chips, while the remaining fractured particles, retained by the particle–matrix interfacial bonding, stay embedded in the matrix, thereby forming a cone-shaped pit on the surface. In Figure 9c,h, debonding between the crystal particles and the matrix occurs during the removal of particle No. 2. At this stage, the cutting path passes through the lower region of the crystal particle. Since the particle exhibits greater strength than both the matrix and the interface, interfacial failure is more likely to occur during cutting, leading to debonding between the particle and the matrix and the formation of a deeper cone-shaped pit. In Figure 9d,i, during the cutting of particle No. 3, the cutting path passes across the top of the crystal particle. Under the combined action of cutting edge shearing and the compression exerted by both the rake and flank faces, the particle fractures. The portion of the fractured crystal particle compressed by the rake face is expelled as chips, while the portion fractured under the flank face remains embedded in the matrix, forming a cone-shaped pit on the surface. In Figure 9e,j, transgranular fracture occurs during the cutting of particle No. 4. Similar to particle No. 3, the cutting path passes across the top of the particle, and the crystal particle is simultaneously subjected to tool edge shearing and compression from both the rake and flank faces. However, in Figure 9j, particle No. 4 not only exhibits transgranular fracture but also undergoes debonding from the matrix. A comparison between particles No. 4 and No. 3 suggests that the smaller particle exhibits a reduced interfacial contact area with the matrix, which may contribute to the occurrence of interfacial debonding under the present cutting condition. Under the larger normal force exerted by the negative rake angle tool, particle No. 4 undergoes both transgranular fracture and interfacial debonding, whereas particle No. 3 mainly experiences transgranular fracture. It should be noted that particle size was not treated as an independent variable in the present study, and therefore its influence on particle removal behavior and pit geometry was not systematically investigated. Previous studies have reported that particle size can affect machining-induced surface damage, where larger particles generally tend to produce larger surface pits after fracture or interfacial debonding, leading to increased surface roughness [48,49,50]. In addition, variations in particle size may alter the interfacial contact area and local stress distribution, thereby influencing the probability of interfacial debonding. These findings from the literature are consistent with the qualitative observations in the present simulations. A systematic investigation of the influence of particle size through dedicated simulations and experiments is beyond the scope of the present study and will be considered in future work.

**Figure 10:**
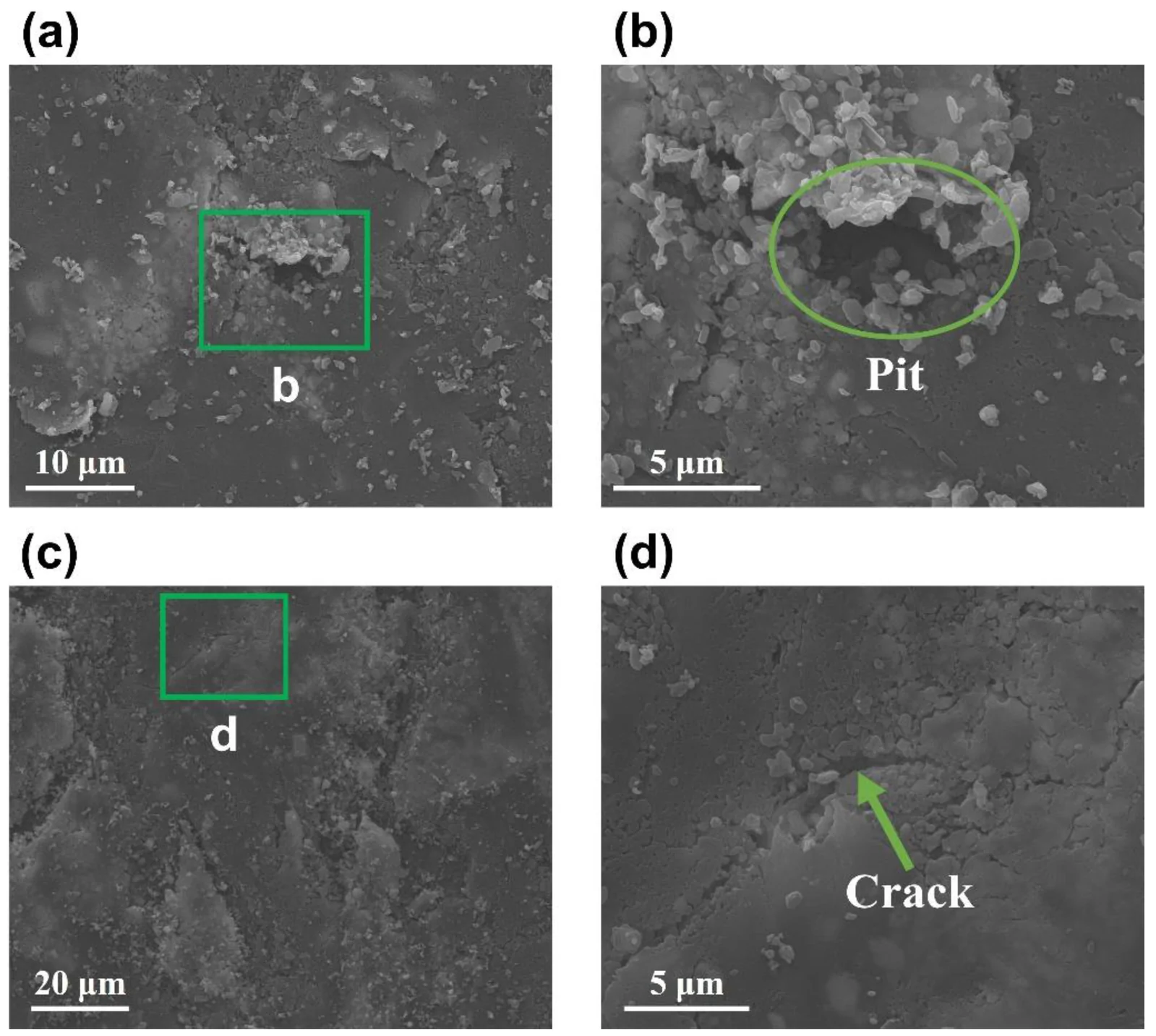
SEM images showing representative surface features on the machined PRPMC surface: (**a**) Surface pit. (**b**) Magnified view of the surface pit shown in (**a**). (**c**) Crack feature. (**d**) Magnified view of the crack feature shown in (**c**).

To further support the discussion of surface pit formation, the machined surface of the PRPMC was examined using a field-emission scanning electron microscope (FE-SEM, JEOL JSM-7800F, JEOL, Akishima, Japan). Figure 10 shows representative SEM images of a surface pit and a crack feature on the machined surface, together with their corresponding magnified views. Specifically, Figure 10a and Figure 10b present the surface pit and its magnified view, respectively, whereas Figure 10c,d show the crack feature and its magnified view. The surface pit and crack feature observed in the SEM images are consistent with those observed in the FE simulations.

### 3.2. Effect of Cutting Depth

**Figure 11:**
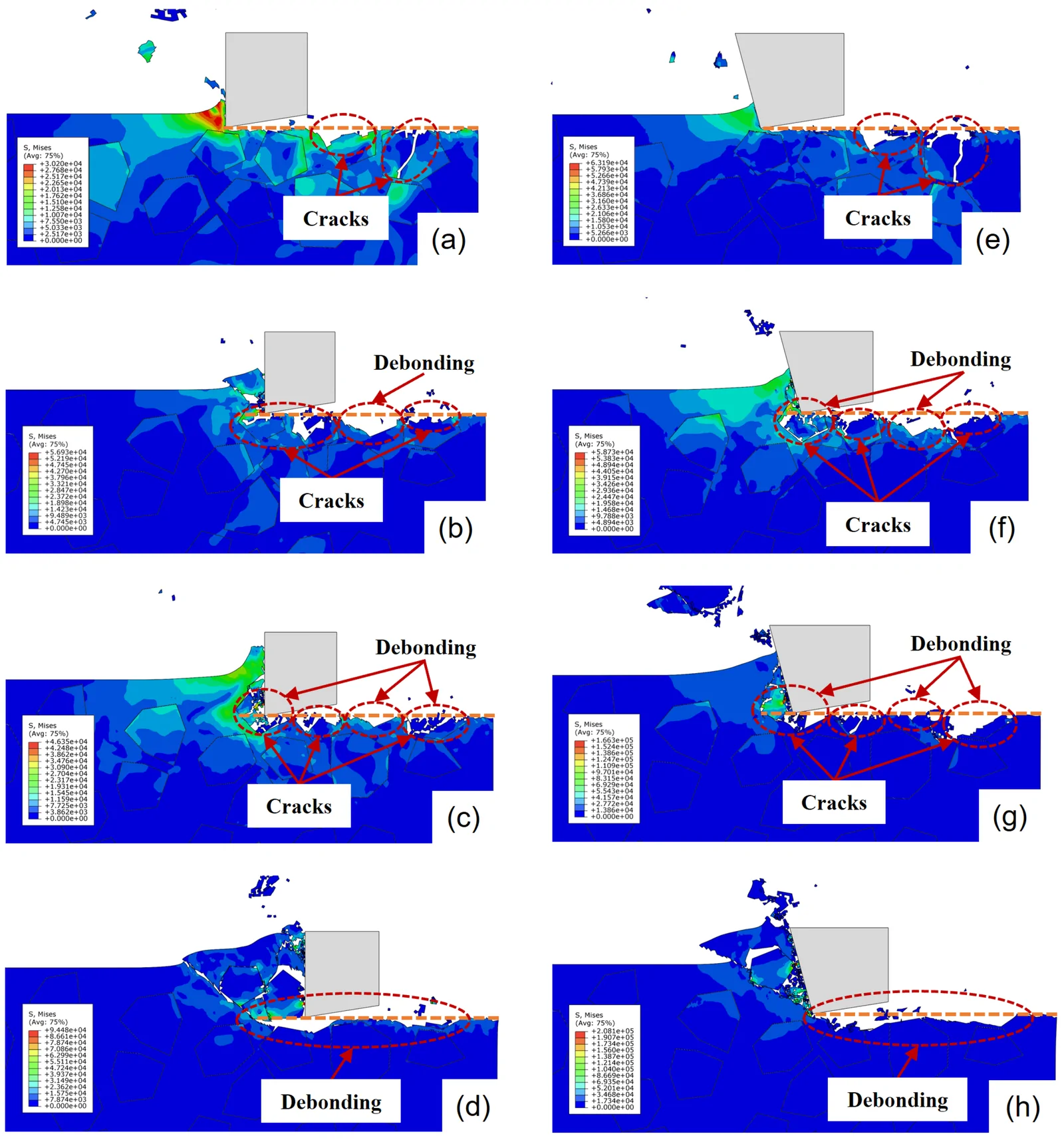
Surface profiles obtained from FE simulations at different cutting depths: (**a**,**e**) 1 μm. (**b**,**f**) 2 μm. (**c**,**g**) 3 μm. (**d**,**h**) 4 μm.

In addition to the cutting depth of 2 μm, FE simulations are also performed for three other cutting depths of 1 μm, 3 μm, and 4 μm. Figure 11 illustrates the surface profiles of PRPMC samples cut with tools of 0° and −15° rake angles at the four different cutting depths. Figure 11a,e show the profiles of PRPMC at a cutting depth of 1 μm, where the pits observed along the cutting path are formed by transgranular fracture of crystal particles. Figure 11b,f correspond to a cutting depth of 2 μm, while Figure 11c,g correspond to a cutting depth of 3 μm, where the pits are attributed to the combined effects of transgranular fracture of crystal particles and debonding between crystal particles and the matrix. Figure 11d,h show the profiles of PRPMC at a cutting depth of 4 μm, where the pits are predominantly caused by debonding between crystal particles and the matrix.

The above results reveal the progressive evolution of pit formation mechanisms with increasing cutting depth. A critical cutting depth of 2 μm is identified, below or equal to which the process corresponds to a small cutting depth and above which it corresponds to a large cutting depth. With increasing cutting depth from the small to the large regime, the dominant pit formation mechanism transitions from transgranular fracture of crystal particles to debonding of crystal particles.

**Figure 12:**
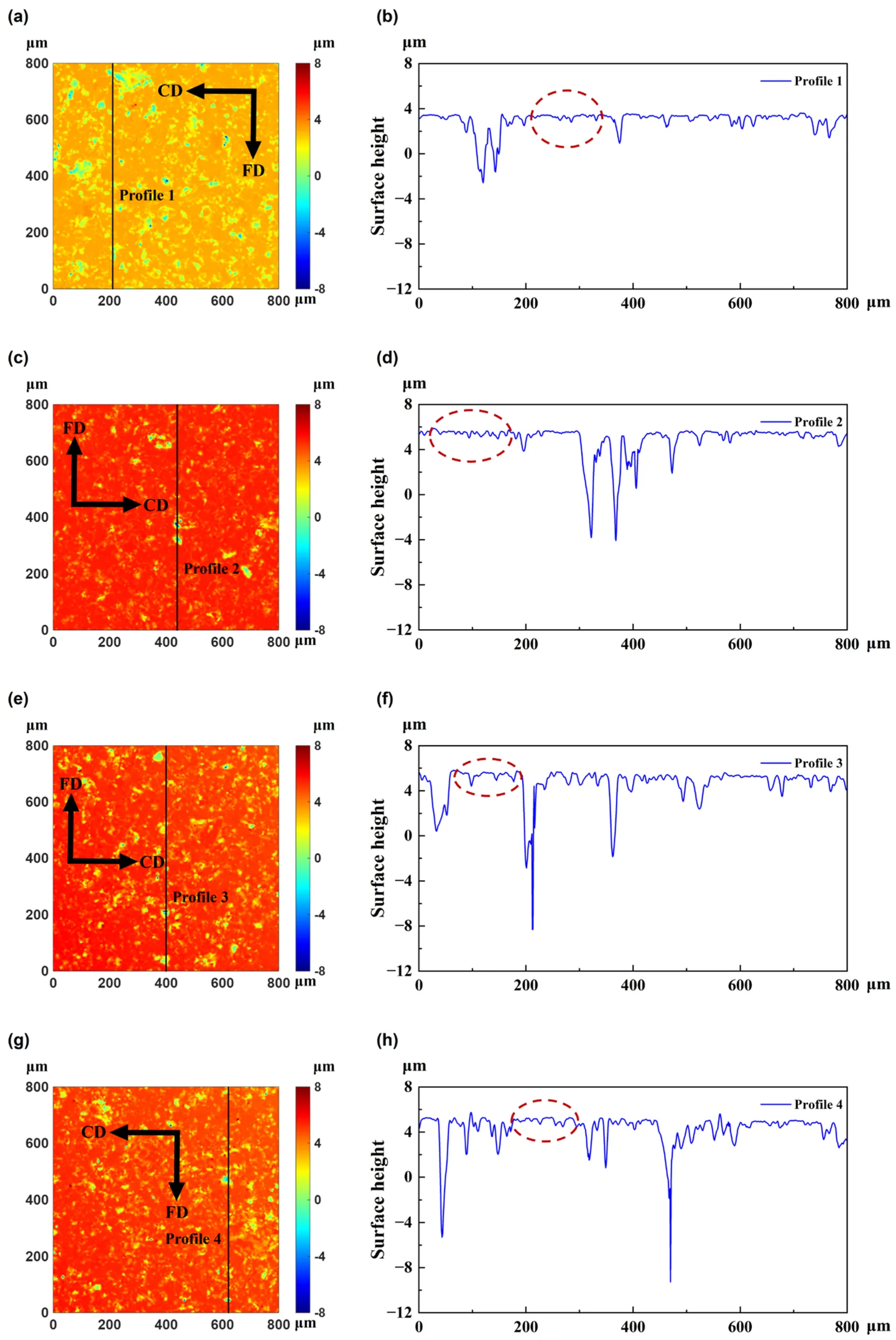
Surface topographies and corresponding two-dimensional profiles of PRPMC after ultra-precision cutting at different cutting depths: (**a**,**b**) 1 μm (Sa 388.11 nm). (**c**,**d**) 2 μm (Sa 439.27 nm). (**e**,**f**) 3 μm (Sa 494.48 nm). (**g**,**h**) 4 μm (Sa 508.37 nm).

Figure 12 presents the surface topographies of the PRPMC after ultra-precision cutting at different cutting depths. As illustrated in Figure 12a, when the cutting depth is 1 μm, pit formation is mainly governed by the transgranular fracture of crystal particles. The arithmetic mean height *S_a_* of the machined surface is 388.11 nm. The corresponding two-dimensional surface profile extracted at this condition is presented in Figure 12b, where only slight fluctuations are observed, indicating that the pits formed by transgranular fracture are relatively shallow. The region marked by the red circle represents the matrix area, showing small amplitude fluctuations, which suggests a minor contribution of plastic side flow. As shown in Figure 12c, when the cutting depth increases to 2 μm, pit formation is still dominated by the transgranular fracture of crystal particles. The corresponding two-dimensional profile in Figure 12d exhibits slightly deeper pits compared with those at a cutting depth of 1 μm. In the matrix region indicated by the red circle, the surface profile shows larger fluctuation amplitudes, suggesting that the plastic side flow of the matrix becomes more pronounced. Consequently, the arithmetic mean height *S_a_* increases to 439.27 nm. With a further increase in the cutting depth to 3 μm, as shown in Figure 12e,f, pit formation becomes dominated by interfacial debonding of the crystal particles, resulting in the formation of deeper pits. In the matrix region indicated by the red circle, the surface profile exhibits significantly larger fluctuations than those observed at cutting depths of 1 μm and 2 μm, and the arithmetic mean height *S_a_* increases to 494.48 nm. As shown in Figure 12g,h, when the cutting depth is further increased to 4 μm, pit formation remains dominated by particle debonding. However, the depth of the pits formed by debonding is comparable to that observed at a cutting depth of 3 μm, and the surface profile fluctuation amplitude in the matrix region differs only slightly. The arithmetic mean height *S_a_* increases slightly to 508.37 nm, which is close to that at 3 μm. Therefore, to achieve a smaller arithmetic mean height *S_a_*, the cutting depth should be appropriately reduced. A smaller cutting depth not only prevents deep pits caused by particle debonding from dominating the surface topography but also reduces the contribution of plastic side flow, thereby further decreasing the surface roughness.

**Figure 13:**
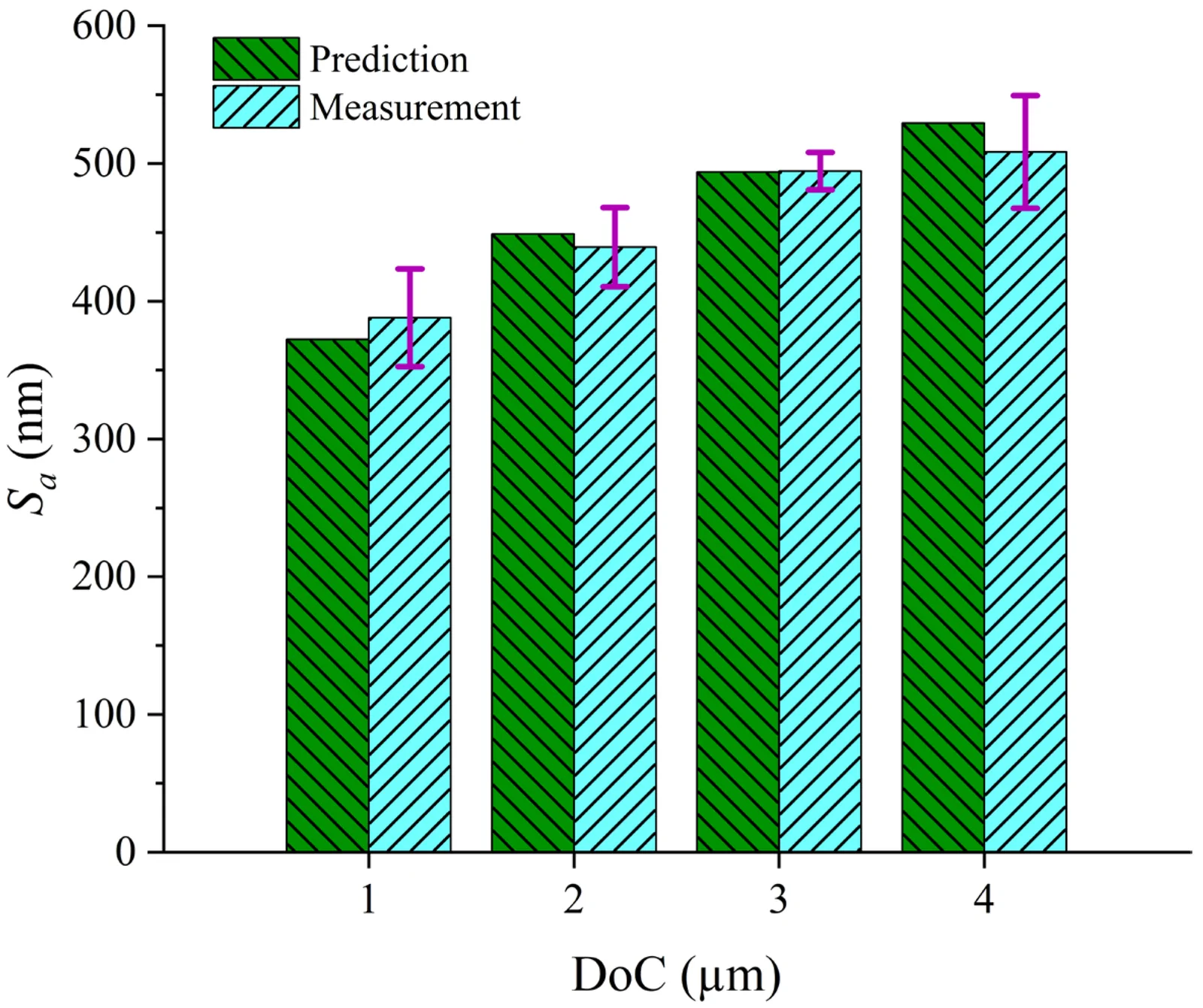
Comparison between the predicted and measured arithmetic mean height Sa at different cutting depths (tool rake angle: 0°, tool nose radius: 1000 μm).

Figure 13 compares the measured results and theoretical model predictions of the surface roughness obtained after ultra-precision cutting with Tool No. 1 at different cutting depths. The results indicate that the arithmetic mean height *S_a_* increases with increasing cutting depth, while the rate of increase gradually decreases. This behavior arises because when the cutting depth exceeds a critical value, pit formation is mainly governed by the interfacial debonding of the crystal particles. The contribution of the pit component to the overall arithmetic mean height *S_a_* then approaches a limiting value, and further increases in *S_a_* are primarily governed by the plastic side flow component rather than the pit component. The maximum relative error between the theoretical predictions and experimental measurements of *S_a_* is 4.11%.

### 3.3. Effect of Tool Rake Angle

**Figure 14:**
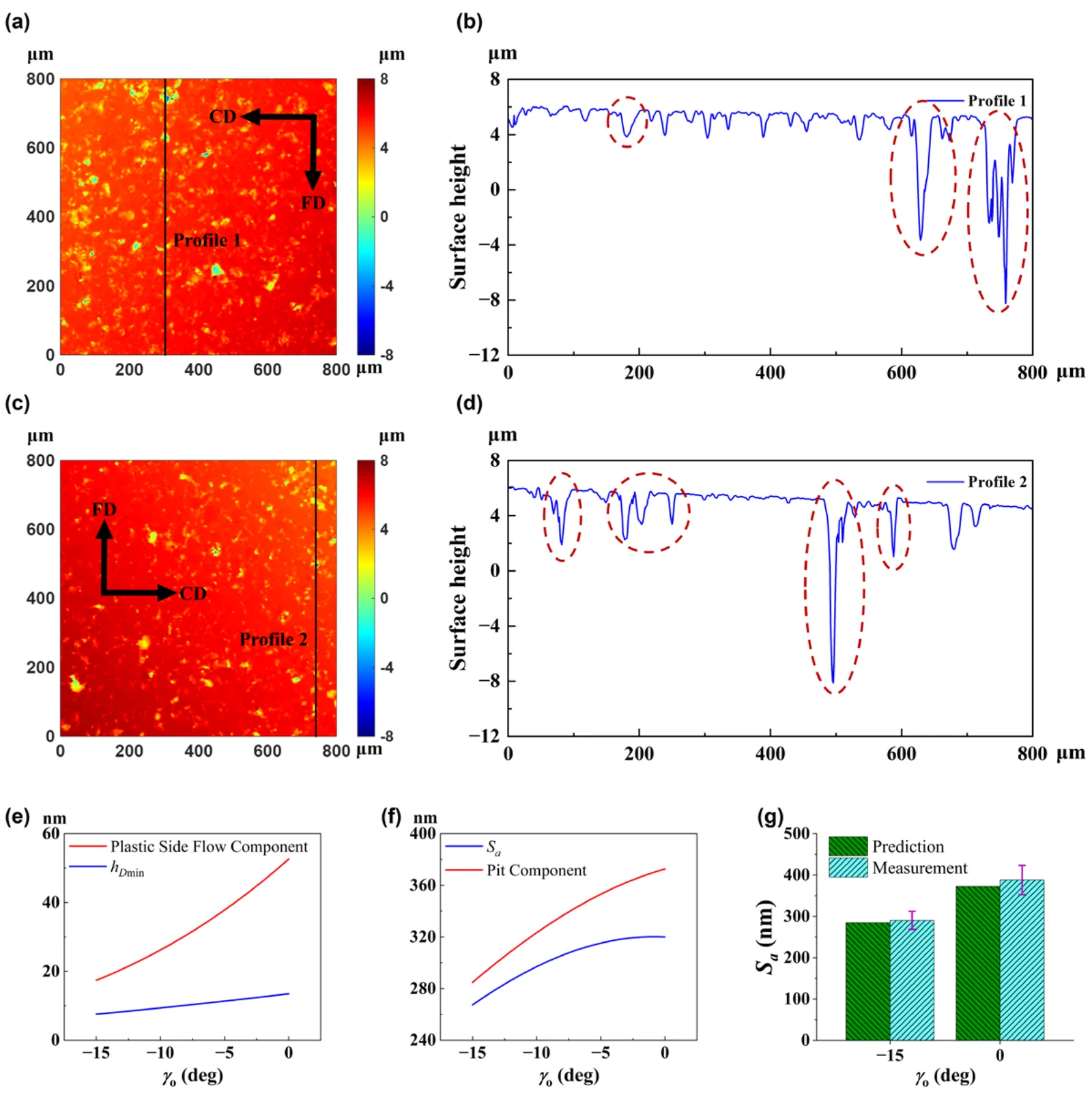
Surface topographies, corresponding two-dimensional profiles, and theoretical predictions of the PRPMC after ultra-precision cutting (cutting depth: 1 μm; tool nose radius: 1000 μm) under different rake angles: (**a**) Surface roughness measurement result at a rake angle of 0° (Sa 388.11 nm). (**b**) Corresponding two-dimensional surface profile at a rake angle of 0°. (**c**) Surface roughness measurement result at a rake angle of −15° (Sa 290.37 nm). (**d**) Corresponding two-dimensional surface profile at a rake angle of −15°. (**e**) Theoretical prediction of the variation in minimum undeformed chip thickness and plastic side flow component with rake angle. (**f**) Theoretical prediction of arithmetic mean height Sa and pit component as functions of rake angle. (**g**) Comparison between predicted and measured arithmetic mean height Sa under different rake angles.

Figure 14 presents the comparison between the measured arithmetic mean height and the theoretical model predictions for the PRPMC machined with tools of different rake angles. Figure 14a presents the surface topography obtained using a tool with a rake angle of 0°, where the arithmetic mean height *S_a_* of the machined surface is 388.11 nm. The corresponding two-dimensional profile, shown in Figure 14b, reveals that the shallow pits are formed by transgranular fracture, while the deeper pits result from interfacial debonding of the crystal particles. Figure 14c displays the surface topography obtained using a tool with a rake angle of −15°, with an arithmetic mean height *S_a_* of 290.37 nm. The corresponding two-dimensional profile is shown in Figure 14d. A comparison between the two-dimensional profiles in Figure 14b,d, particularly in the regions indicated by the red circles, shows that the machined surface obtained with the −15° rake angle exhibits larger height fluctuations, indicating that the pits formed by transgranular fracture or interfacial debonding are deeper. However, the distance between the large fluctuations is smaller, suggesting that the pit diameters are reduced, which agrees well with the theoretical model. Although the pits formed by the −15° rake angle tool are deeper, their smaller diameters result in a reduced overall pit volume.

Figure 14e illustrates the variation in minimum undeformed chip thickness and plastic side flow component with tool rake angle when the cutting edge radius and the chip–tool friction coefficient are constant. As the tool rake angle increases from −15° to 0°, the minimum undeformed chip thickness rises from 7.6 nm to 13.5 nm, and the plastic side flow component increases from 17.4 nm to 52.6 nm. According to Liu et al. [28] and He et al. [24], stress concentration is one of the primary causes of plastic side flow. As the tool rake angle increases from −15° to 0°, the degree of stress concentration in the material during cutting increases, leading to a further enhancement of the plastic side flow component. Figure 14f presents the variations in the arithmetic mean height *S_a_* and the pit component with the tool rake angle. The pit component initially increases and then decreases as the tool rake angle rises from −15° to 0°, reaching a maximum of 320.1 nm at a rake angle of -0.86°. In contrast, the arithmetic mean height *S_a_* increases monotonically within this rake angle range because the increase in the plastic side flow component compensates for the reduction in the pit component. Figure 14g compares the measured and theoretical results for Tool No. 1 and Tool No. 2 at a cutting depth of 1 μm. The results indicate that the maximum relative error between the theoretical predictions and experimental measurements of *S_a_* is 4.07%.

### 3.4. Effect of Tool Nose Radius

**Figure 15:**
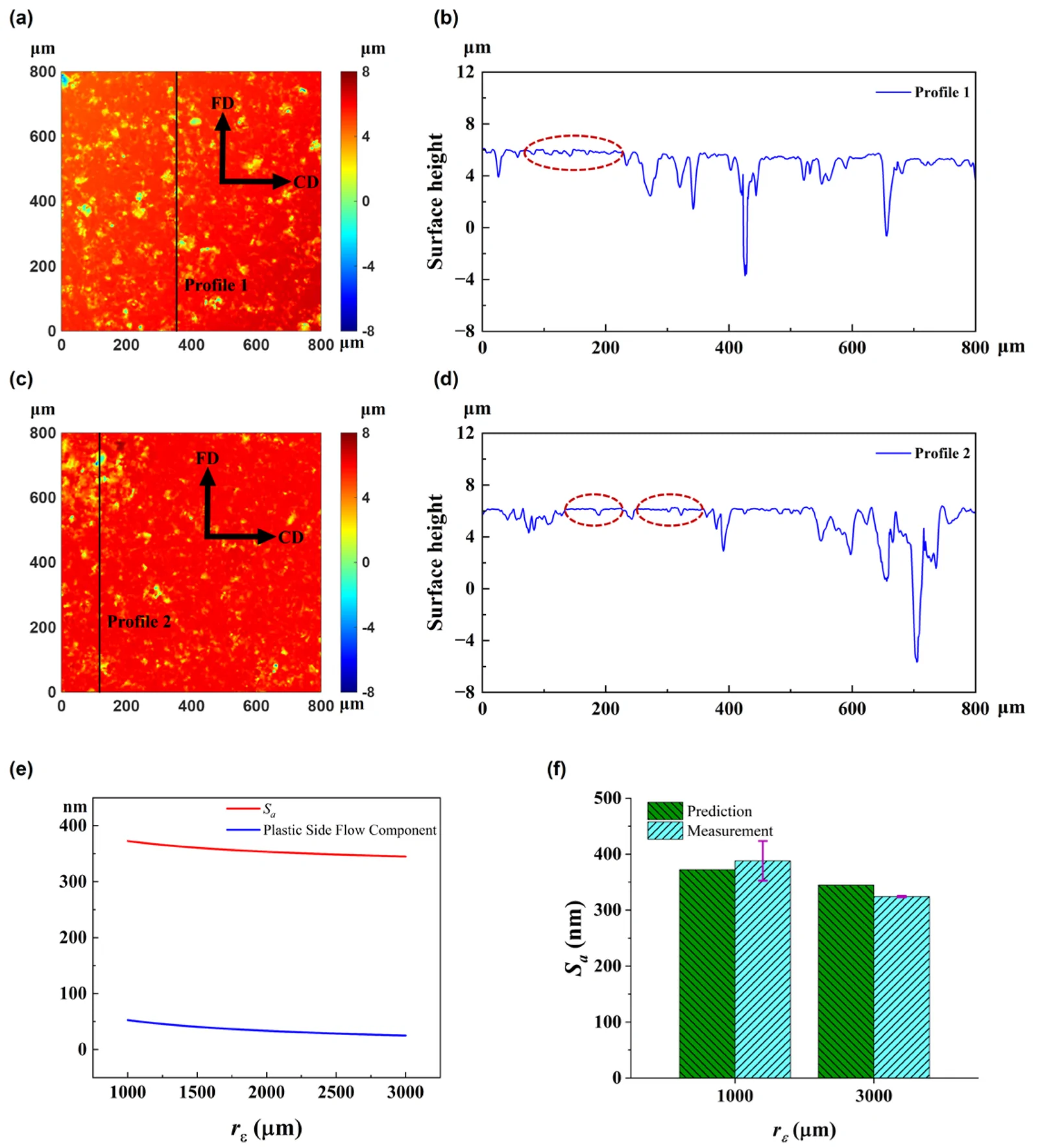
Surface topographies, corresponding two-dimensional profiles, and theoretical predictions of the PRPMC after ultra-precision cutting (cutting depth: 1 μm; tool rake angle: 0°) under different tool nose radii: (**a**) Surface roughness measurement result with a tool nose radius of 1000 μm (Sa = 388.11 nm). (**b**) Corresponding two-dimensional surface profile at a tool nose radius of 1000 μm. (**c**) Surface roughness measurement result with a tool nose radius of 3000 μm (Sa = 324.22 nm). (**d**) Corresponding two-dimensional surface profile at a tool nose radius of 3000 μm. (**e**) Theoretical prediction of the arithmetic mean height Sa and plastic side flow component as functions of tool nose radius. (**f**) Comparison between predicted and measured arithmetic mean height Sa under different tool nose radii.

Figure 15 presents the measured and theoretically predicted arithmetic mean height values obtained from cutting PRPMC with tools of different tool nose radii. Figure 15a and Figure 15c present the machined surface topographies for tool nose radii of 1000 μm and 3000 μm, respectively, with the measured arithmetic mean height *S_a_* values of 388.11 nm and 324.22 nm. The corresponding two-dimensional surface profiles are shown in Figure 15b,d, with the matrix regions indicated by red circles. A comparison between the red circled areas in Figure 15b,d indicates that the surface undulations are smaller when using the tool with a 3000 μm nose radius, implying a reduced degree of plastic side flow within the matrix. Figure 15e illustrates the theoretical predictions of the arithmetic mean height and plastic side flow component as a function of tool nose radius. As the tool nose radius increases, the enlarged tool–workpiece contact area distributes the contact stress more uniformly, reducing local stress concentration. Consequently, matrix plastic deformation and plastic side flow are suppressed [51], leading to a decrease in the arithmetic mean height *S_a_*. According to Equation (17), the tool nose radius also influences the theoretical residual height component. However, as shown in Equations (30) and (31), the residual height component *S_ath_* and the plastic side flow component *w* can be calculated separately. The contribution of each component was determined by dividing the corresponding component by the total predicted surface roughness. The results indicate that the residual height component contributes approximately 0.01% to *S_a_*, whereas the plastic side flow component contributes about 17%. Hence, the influence of tool nose radius on the arithmetic mean height is dominated by the variation in the plastic side flow component [52]. Figure 15f compares the measured and predicted *S_a_* values for cutting with tool nose radii of 1000 μm and 3000 μm, revealing a maximum relative error of 6.33% between the experimental measurements and theoretical predictions.

### 3.5. Experimental Measurements and Theoretical Predictions

Ten groups of ultra-precision cutting experiments were conducted under different processing parameters, and the experimental measurements along with the theoretical model predictions are presented in Table 8. The maximum relative error between the theoretical predictions and the experimental results is 9.20%, while the average relative error is 4.66%. These results indicate that the theoretical model can accurately predict the influence of tool rake angle, tool nose radius, and cutting depth on the arithmetic mean surface roughness. To further assess the applicability of the proposed model under extremely small cutting depths, an additional cutting experiment was conducted using Tool No. 1 at a cutting depth of 0.9 μm. The measured *S_a_* was 362.16 nm, whereas the predicted value was 356.20 nm, corresponding to a relative error of 1.65%. This result indicates that the proposed model remains applicable at cutting depths below 1 μm.

## 4. Conclusions

In this study, finite element simulations, theoretical modeling, and ultra-precision cutting experiments were combined to investigate surface pit formation and surface roughness evolution during the ultra-precision cutting of a PRPMC. The main conclusions are as follows:

(1) Surface pits originate from the fracture and debonding of crystal particles during cutting. Particle fracture tends to produce relatively shallow pits, whereas particle–matrix interfacial debonding results in deeper surface defects.
(2) Cutting depth significantly influences the pit formation mechanism. As the cutting depth increases, the dominant removal mode gradually shifts from particle fracture to interfacial debonding, leading to the formation of larger and deeper pits.
(3) A predictive model for arithmetic mean height was established based on particle removal behavior and pit formation characteristics. The model successfully captures the effects of tool rake angle, tool nose radius, and cutting depth on surface roughness, with good agreement between theoretical predictions and experimental measurements.

These findings provide insight into the surface generation mechanism of PRPMCs and offer guidance for the optimization of ultra-precision cutting processes.

## Figures and Tables

**Table 1:** Simulation parameters used in FE model.

Mesh Type	Mesh Size (nm)	Cutting Speed (mm/s)	Cutting Depth (μm)
CPE4R	200	400	1, 2, 3, 4

**Table 2:** The Johnson–Cook constitutive parameters of matrix [34,35,36,37,38,39].

Constitutive Parameters		Failure Parameters	
*A* (MPa)	15	*d*_1_	0.1
*B* (MPa)	20	*d*_2_	0.4
*C*	0.002	*d*_3_	0.1
*n*	0.5	*d*_4_	0.005
*m*	1.4	*d*_5_	0

**Table 3:** Physical parameters of matrix [34,35,36,37,38,39].

Density (g/cm^3^)	Elastic Modulus (MPa)	Poisson’s Ratio	Failure Displacement
2.02	5.5	0.33	0.11

**Table 4:** Constitutive parameters for brittle fracture of barium sulfate [34,35,36,37,38,39,40,41].

Density (g/cm^3^)	Elastic Modulus (GPa)	Poisson’s Ratio	Fracture Stress (MPa)	Fracture Strain	Failure Displacement
1.938	25.3	0.1	80	0.008	0.003

**Table 5:** Cohesive element parameters of matrix– interface [35,36,37,38,39].

*k_n_* (GPa)	*k_t_* (GPa)	${\bar{t}}_{n}$ (MPa)	${\bar{t}}_{t}$ (MPa)	Failure Displacement
5.5	2.5	22	11	0.04

**Table 6:** Parameters of natural single-crystal diamond tools.

Tool No.	Rake Angle (deg)	Flank Angle (deg)	Tool Nose Radius (μm)
1	0	9	1000
2	−15	9	1000
3	0	9	3000

**Table 7:** Cutting conditions for face turning tests with natural single-crystal diamond tools.

Experiment No.	Tool No.	Spindle Speed (rpm)	Feed Rate (μm/r)	Cutting Depth (μm)
1	1	1000	1	1
2	1	1000	1	2
3	1	1000	1	3
4	1	1000	1	4
5	2	1000	1	1
6	2	1000	1	4
7	3	1000	1	1
8	3	1000	1	2
9	3	1000	1	3
10	3	1000	1	4

**Table 8:** Comparison between measured and predicted *S_a_* values.

Experiment No.	Tool No.	Cutting Depth (μm)	Measurement (nm)	Prediction (nm)	Error (%)
1	1	1	388.11	372.31	4.07
2	1	2	439.27	448.74	2.15
3	1	3	494.48	493.82	0.13
4	1	4	508.37	529.26	4.11
5	2	1	290.37	284.63	1.98
6	2	4	367.69	334.04	9.15
7	3	1	324.22	344.74	6.33
8	3	2	346.45	378.31	9.20
9	3	3	407.55	398.11	2.32
10	3	4	445.70	413.67	7.19

## Data Availability

The data that support the findings of this study are available from the corresponding author upon reasonable request.

## Nomenclature

*a*	Primary crack length (µm)
*ap*	Cutting depth (µm)
*c*	Pit radius (µm)
*c*_1_, *c*_2_	Material-related constants for Hertzian contact
*d*	Indentation depth of the rigid indenter
*d*_1_–*d*_5_	Failure parameters
*f*	Feed rate (µm/r)
*h*	Pit depth (µm)
*h*_*D*min_	Minimum undeformed chip thickness (nm)
*i*	Number of pits in the sampling area
*j*	Number of feeds within the sampling length
*k*_1_–*k*_4_	Material-dependent correction coefficients
*k_n_*	Normal stiffness of interface
*k*_r_	Correction factor for tool nose radius
*k_t_*	Tangential stiffness of interface
*k*_D_	Correction factor for cutting depth
*l*_1_	Sampling length along the feed direction (µm)
*l*_2_	Sampling length along the cutting speed direction (µm)
*m*	Thermal softening index
*n*	Strain hardening coefficient
*p*	Contact stress (MPa)
*p*_0_	Maximum contact stress (MPa)
*r*	Curvature radius of the rigid indenter
*r*_n_	Cutting edge radius (nm)
*r_ε_*	Tool nose radius (µm)
${\bar{t}}_{n}$	Normal nominal stress of interface (MPa)
${\bar{t}}_{t}$	Tangential nominal stress of interface (MPa)
*v*	Cutting speed (mm/s)
*w*	Plastic side flow component (nm)
*α*_o_	Flank angle of diamond tool (deg)
*β*	Friction angle between diamond tool and work material (deg)
*γ*_o_	Rake angle of diamond tool (deg)
*θ*	Angle between the primary crack and the free surface (deg)
*μ*	Friction coefficient between diamond tool and work material
*σ*	Normal stress (MPa)
*ν*	Poisson’s ratio
*ϕ*	Shear angle (deg)
*χ*	Material properties related to Poisson’s ratio
*A*	Initial yield strength (MPa)
*B*	Strain hardening modulus (MPa)
*C*	Strain rate sensitivity coefficient
*F*	Indenter pressure (mN)
*F*_P_	Horizontal cutting force (mN)
*F*_Q_	Normal cutting force (mN)
*F*_C_	Frictional force on the chip (mN)
*K*_I_	Mode I stress intensity factor (MPa∙mm^0.5^)
*K*_IC_	Mode I fracture toughness (MPa∙mm^0.5^)
*K*_II_	Mode II stress intensity factor (MPa∙mm^0.5^)
*K*_IIC_	Mode II fracture toughness (MPa∙mm^0.5^)
*N*c	Compressive force on the chip (mN)
*R*	Resultant cutting force (mN)
*R_tew_*	Theoretical residual height (nm)
*Rth*	Residual height (Spanzipfel formula) (nm)
*Rth__rev_*	Residual height (modified Spanzipfel formula) (nm)
*S*	Sampling area (µm^2^)
*S_a_*	Arithmetic mean height (nm)
*S_ath_*	Residual height component (nm)
*V*_1_	Volume of a single surface pit (µm^3^)
*V*_2_	Volume of residual profile along feed direction (µm^3^)
